# Factors influencing the growth hormone peak and plasma insulin-like growth factor I in young adults with pituitary stalk interruption syndrome

**DOI:** 10.1186/1472-6823-8-7

**Published:** 2008-07-11

**Authors:** Mariana Marcu, Christine Trivin, Jean-Claude Souberbielle, Raja Brauner

**Affiliations:** 1 Université Paris Descartes and Assistance Publique Hôpitaux de Paris, Hôpital Bicêtre, Unité d'Endocrinologie Pédiatrique, 94270 Le Kremlin Bicêtre, France; 2 Assistance Publique Hôpitaux de Paris, Hôpital Necker-Enfants Malades, Service d'Explorations Fonctionnelles, 75743 Paris, France

## Abstract

**Background:**

The diagnostic criteria for growth hormone (GH) deficiency (GHD) in adolescents and young adults are not yet clearly established.

We evaluated the factors influencing the GH peak and plasma insulin-like growth factor (IGF) I in order to determine the cut-off limits for the diagnosis of GHD during the transition period.

**Methods:**

21 patients treated for GHD due to pituitary stalk interruption syndrome at 5.7 ± 4.1 years were reevaluated at 16.0 ± 1.8 years, 0.6 ± 0.6 years after the end of GH treatment. Group 1 had isolated GHD (n = 9) and group 2 had multiple pituitary deficiencies (n = 12), including deficiencies of thyroid stimulating (n = 12), adrenocorticotropin (n = 8) and gonadotropin (n = 9) hormones.

**Results:**

At diagnosis, group 1 had a greater pituitary height (2.8 ± 1.2 vs 1.6 ± 1.1 mm, P = 0.03) and GH peak (3.8 ± 1.9 vs 1.6 ± 1.5 ng/ml, P < 0.02) than did group 2.

At last evaluation, group 1 had greater GH peak (3.9 ± 1.9 vs 0.2 ± 0.4 ng/ml, P = 0.0001) and plasma IGF I (211 ± 88 vs 78 ± 69 ng/ml, P < 0.002) than did group 2. No group 1 and 9 group 2 patients had an undetectable GH peak, while the 3 others had GH peak below 1 ng/ml.

The GH peak decreased between diagnosis and last evaluation only in group 2 (P < 0.008).

**Conclusion:**

The GH peak response to pharmacological stimulation and the plasma IGF I concentration in young adults with GHD of childhood onset depend on the presence of additional pituitary deficiencies, reflecting a more severe defect of the hypothalamic-pituitary axis. The sex steroids cannot increase the IGF I if the GH secretion is zero.

## Background

The diagnostic criteria for growth hormone (GH) deficiency (GHD) in adolescents and young adults, defined as the transition period, are not yet clearly established. Severe GHD in adults is defined by a peak GH response to hypoglycemia of less than 3 ng/ml [1], while a limit of 5 ng/ml in response to a stimulation test has been proposed for adolescents in transition to adult care [2].

The factors reported to influence the GH peak and plasma insulin-like growth factor (IGF) I concentration in GHD are the age at onset (childhood or adulthood) [3] and the etiology (hypothalamic-pituitary lesion, cranial irradiation or idiopathic) [4]. However, the effect of the spontaneous pubertal increase in sex steroids at puberty on these two parameters in GH-deficient patients has not been clearly evaluated [2].

We analysed longitudinally 21 patients with pituitary stalk interruption syndrome (PSIS). We classified them as isolated GHD and multiple pituitary deficiencies. We compared the GH stimulated peak and plasma IGF I concentrations of the two groups and at diagnosis before puberty and after their growth had ended. Our objective was to evaluate the factors influencing the GH peak and plasma IGF I in order to determine the cut-off limits for the diagnosis of GHD during the transition period.

## Methods

### Patients

This retrospective longitudinal study included 21 consecutive patients (13 boys and 8 girls) monitored by one of us (R Brauner) in a tertiary university pediatric hospital. All had GHD of prepubertal onset due to PSIS, had reached their adult height and were reevaluated for their GH secretion after the end of GH treatment. The criterion for diagnosing GHD during childhood was a GH peak response (maximal GH concentration) of less than 7 ng/ml after 2 stimulation tests, excluding the GH-releasing hormone (GHRH) test. Other features suggesting GHD were microphallus (6 boys), hypoglycemia (n = 8) and other hypothalamic-pituitary deficiencies (n = 12).

PSIS was diagnosed on the basis of no visible pituitary stalk, no normal posterior lobe hypersignal in the sella turcica, and the presence of a hyperintense nodule in the region of the infundibular recess of the third ventricle [5].

The patients were divided into two groups: group 1 with isolated GHD (n = 9) and group 2 with multiple pituitary deficiencies (n = 12), including deficiencies of thyroid stimulating (n = 12), adrenocorticotropin (n = 8) and gonadotropin (n = 9) hormones.

The ages were 5.7 ± 4.1 years at diagnosis and 16.0 ± 1.8 years at the last GH evaluation, 0.6 ± 0.6 years after the end of GH treatment.

### Protocol

The patients and their parents were informed that the evaluation would be performed to measure GH secretion, to adjust the replacement therapy (other than GH), and to prepare for the transfer of the follow-up to adult departments. They gave their consent for this evaluation. All patients underwent 3 stimulation tests, 2 at diagnosis before GH treatment and the third after treatment had ended. Each evaluation was performed in a single morning with patients in a fasting state, and included a physical examination plus measurements of height and weight. Before treatment, at least 1/2 stimulations used arginine insulin (n = 15) or glucagon (n = 5), except for 1 patient who was stimulated with ornithine. The larger peak was used for statistical analysis. GH therapy had been completed for at least one month before the third evaluation, but the other hormone replacement therapies were continuing (Levothyrox 75–100 microg/m^2^/d, hydrocortisone 10 mg/m^2^/d, ethinyl estradiol or testosterone, see below). The stimulations used at the third test were arginine insulin (n = 9), glucagon (n = 7), or ornithine (n = 5). Blood samples were obtained at 08.00 h for measuring free thyroxin, cortisol, testosterone or estradiol. The cortisol concentrations were not measured in the patients on hydrocortisone replacement therapy. Plasma IGF I was measured in all but 3 at diagnosis and in all at the third evaluation.

The patients with gonadotropin deficiency had no pubertal development despite being of pubertal chronological and bone ages and no gonadotropin response to a gonadotropin releasing hormone stimulation test. The girls had been given oral ethinyl estradiol (2 microg/day) from the age of around 12–13 years, and the boys testosterone heptylate (25 mg i.m. every 14 days) from the age of around 13–14 years.

### Methods

Height was measured twice with a Harpenden stadiometer. The height and body mass index (BMI, weight in kg/height in m squared) are expressed as SDS for chronological age [6,7]. Commercial immunoassays were used to measure GH and IGF I (IGF-I-RIACT, Cis Bio, Gif sur Yvette, France). GH was measured over the years using different immunoassays calibrated against different reference preparations. GH peaks were recalculated in order to be expressed in ng/ml of the international reference standard 98/574 (recombinant 22 kDa GH, 1 ng = 3 microU). The control group for plasma IGF I concentrations at the first evaluation included normal prepubertal children, and at the third evaluation it included 31 adolescents aged 14–16 years and 30 young adults aged 17–20 years with normal height and weight and spontaneous pubertal development [8].

Data are expressed as means ± SD. Groups were compared with the Kruskall Wallis test followed by a Mann-Whitney U tests and repeated measures were compared with the Wilcoxon rank test.

## Results

At diagnosis, all patients were prepubertal (Additional file 1). The oldest patients had bone ages of 11 years (case 9) and 10 years (case 21). The 2 groups had similar chronological ages, heights, BMI, GH peaks after GHRH test, plasma IGF I (expressed in SDS) and prolactin concentrations (11.2 ± 8.4 vs 13.0 ± 7.8 ng/ml). Group 1 had significantly greater pituitary heights and GH peaks than did group 2.

At the last evaluation, the group 1 patients had greater GH peaks and more plasma IGF I (expressed both in ng/ml and in SDS) than did those in group 2. No group 1 and 9 group 2 patients had an undetectable GH peak, while the 3 others had GH peak below 1 ng/ml. The two group 2 patients with IGF I concentrations similar to those of the group 1 patients were girls (cases 18 and 19) with GH peaks of 0.9 ng/ml and spontaneous puberty. The plasma testosterone in boys and estradiol in girls at the third evaluation were pubertal in group 1 and low, but varying, in the group 2 patients evaluated on low dose replacement therapy.

The GH peaks and plasma IGF I concentrations (SDS) were correlated at diagnosis (P < 0.04) and at the last evaluation (P < 0.001).

**Figure 1:**
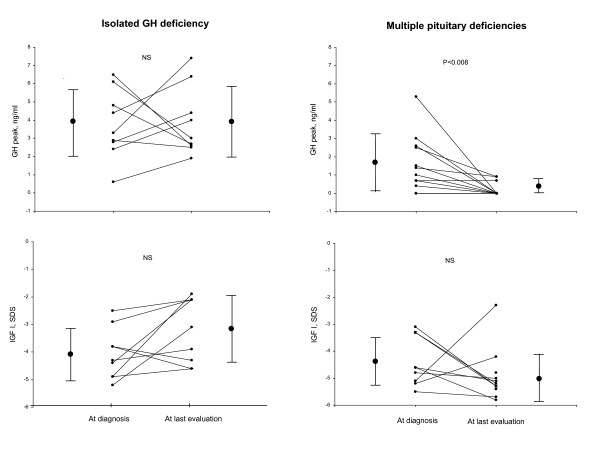
Data for each of 21 patients with GH deficiency with pituitary stalk interruption syndrome at diagnosis and after GH treatment classified according to whether there was isolated GHD (n = 9) or multiple.

The GH peak decreased between diagnosis and the last evaluation only in group 2 (P < 0.008, Table 1 and Fig 1).

**Table 1:** Comparison between before and after GH treatment

Groups (n)	Isolated GH deficiency (9)		P	Multiple pituitary deficiencies (12)		P
				
	at diagnosis	at last evaluation		at diagnosis	at last evaluation	
Height, SDS	-3.0 ± 1.0	-1.0 ± 0.9	<0.008	-3.1 ± 1.6	-0.9 ± 0.7	<0.004
BMI, SDS	-0.3 ± 1.2	0.6 ± 1.4	0.03	0.4 ± 2.4	0.6 ± 1.7	NS
GH peak, ng/ml	3.8 ± 1.9	3.9 ± 1.9	NS	1.6 ± 1.5^a^	0.2 ± 0.4^b^	<0.008
IGF I, SDS	-4.1 ± 0.9	-3.2 ± 1.2	NS	-4.4 ± 0.9	-5.0 ± 0.9^c^	NS
Testosterone, ng/ml		5.3 ± 1.8			1.7 ± 2.0	

Mean ± SD

Isolated GH deficiency compared to multiple pituitary deficiencies: a: P < 0.02; b: P = 0.0001; c: P < 0.002

## Discussion

The GH peak response to pharmacological stimulation and the plasma IGF I concentration in young adults with GHD of childhood onset depend on the presence of additional pituitary deficiencies, reflecting a more severe defect of the hypothalamic-pituitary axis. Sex steroids cannot increase the IGF I if the GH secretion is zero.

The key features of this study are: 1) all the patients had GHD due to PSIS of prepubertal onset; 2) each of them was evaluated longitudinally before puberty and as a young adult and the data from these evaluations were compared.

### 1. Effect of the association with other pituitary deficiencies

The group 1 patients with isolated GHD had significantly greater pituitary height and GH peaks at diagnosis before puberty than did the group 2 patients with multiple deficiencies, despite their similar ages, heights and BMI. This may be due to differences in the way PSIS occurred in the 2 groups. We reported a positive correlation between the GH peak after GHRH and the anterior pituitary height, GH peak after no GHRH stimulation and the spontaneous GH peak in patients with GHD (and PSIS in 22/28) [9]. While the GH peak after GHRH was greater in group 1 than in group 2 in the present study, the difference was not significant, possibly because only three group 1 patients underwent the GHRH test.

This study confirms that the GH peak response to pharmacological stimulation in GHD during childhood and in young adults depends on the presence of additional deficiencies. Maghnie et al [10] reported that all the 13 patients with PSIS tested as young adults had GH peak responses to arginine, insulin and sequential tests that were below 3 ng/ml, while 4 out of 21 patients of the present study, all with isolated GHD, had a GH peak greater than 3 ng/ml. Their findings and our results may differ because more of our patients had isolated GHD. Hartman et al [11] found that 41% of their adult patients without pituitary hormone deficiencies other than GH had a GH peak < 2.5 ng/ml, while 67% lacking one other pituitary hormone, 83% of those lacking two pituitary hormones, 96% of those lacking three pituitary hormones, and 99% of those lacking four pituitary hormones had a GH peak < 2.5 ng/ml. Their 3 patients with idiopathic GHD and a GH peak > 2.5 (2.9, 10 and 18) ng/ml despite three deficiencies had GHD of adult onset and the 2 women were given oral estrogen.

### 2. Effect of sex steroids on GH-IGF I

The group 1 patients with spontaneous puberty showed no increase in the GH peak in response to sex steroid secretion, but the plasma IGF I concentrations (ng/ml) did increase. The variations in the plasma estradiol or testosterone concentrations in the group 2 patients at the last evaluation probably partly reflect differences in the interval between the last administration of ethinyl estradiol or testosterone and the GH evaluation in those with gonadotropin deficiency. Among the three group 2 patients with spontaneous puberty, two had plasma IGF I concentrations similar to those of group 1 patients, while the third (case 12) had a very low plasma IGF I despite a plasma testosterone concentration of 5.6 ng/ml. They differed in that their GH peak was greater than zero in the first two (cases 18 and 19) and undetectable in the third patient (case 12). This suggests that some residual GH secretion is necessary for the sex steroids to increase IGF I. This probably explains the data reported by Aguiar-Oliveira et al [12], who studied patients with a mutated GHRH receptor that was responsible for a GH peak of 0.01–0.2 ng/ml. They were surprised to find that there was no significant pubertal rise in IGF I, IGF II, IGF binding protein-3, or acid-labile subunit concentrations, as pubertal development was normal, although slightly delayed.

The administration of high doses of sex steroids before the last evaluation to the patients with gonadotropin deficiency, to obtain levels similar to those of group 1, would help to confirm the absence of a direct affect of sex steroids on IGF I. However, one patient from group 1 (case 3) and two from group 2 (cases 10 and 15) with similar plasma testosterone concentrations (spontaneously or after administration) but different GH peaks (3 and 0 ng/ml) had different plasma IGF I concentrations (296 vs 59 and 78 ng/ml). Martinez et al [13] evaluated the effect of estradiol priming on the GH-IGF axis in 15 patients with GHD and radiological findings after magnetic resonance imaging. They were given a daily dose of 1 or 2 mg micronized estradiol or placebo for 3 days before a sequential arginine-clonidine test. Estradiol did not significantly stimulate GH secretion (3.1 ± 2.4 vs 4.5 ± 2.7 ng/ml). The IGF I concentrations of 14/15 patients on placebo were below normal and estradiol did not change the mean of the group (28 ± 48 vs 25 ± 29 ng/ml).

### 3. Diagnosis of GHD in adults

The key feature of this study is that all the patients had GHD due to PSIS of prepubertal onset. The majority of the patients with adult-onset GHD had had a hypothalamic-pituitary lesion and had been treated by surgery and/or irradiation. Their results are consistent with previous finding that patients with craniopharyngioma [8,14] or given low dose cranial irradiation [4] may have a normal plasma IGF I concentration. This may partly explain why Hoffman et al [15] found that 70% of the IGF I and 72% of the IGF binding protein-3 concentrations were within the normal range in adults with a GH peak below 5 ng/ml after an insulin test (pituitary adenoma or cranial irradiation) while de Boer et al [16] found only 4% of the IGF I and 8% of the IGF binding protein-3 concentrations were normal in young adults with idiopathic GHD of childhood onset.

Our results partly explain the difficulty of defining a limiting plasma IGF I concentration for diagnosing GHD in adults. Hartman et al [11] concluded that patients with an appropriate clinical history and having 3 or 4 additional hormonal deficiencies or a serum IGF I less than 84 ng/ml do not require a GH stimulation test for the diagnosis of adult GHD. We found that IGF I was very low in all patients with gonadotropin deficiency, and in the sole patient with spontaneous puberty but a GH peak of 0 ng/ml.

### 4. Analysis of the limitations of the study

The number of subjects studied is limited, but there are no reported data on the longitudinal evolution in GH and IGF I in patients with PSIS and only limited data on patients with PSIS in the transition period [17]. These authors used the GHRH plus arginine test. In the present study, the GH peaks for a given patient obtained during childhood and as a young adult were not obtained using similar stimulations, but arginine insulin or glucagon were used as stimulus in the majority of patients. The short time between stopping GH and testing our patients may partly explain the concentrations of IGF I. Thus, Maghnie et al [10] reported that the IGF I concentrations decreased significantly 6 and 12 months after stopping GH in patients with PSIS. However, the interval was greater than one month in all. Group 2 patients had low plasma IGF I concentrations, whatever the interval. A recent Endocrine Society Clinical Practice Guideline [18] suggested that the interval between the reevaluation and the discontinuation of GH treatment should not be less than one month, and because of the irreversible nature of GHD in children with PSIS and multiple hormonal deficiencies, a low IGF I measured at least one month after discontinuing treatment is sufficient documentation of persistant GHD without additional provocative testing. Our data confirm this statement.

## Conclusion

The GH peak response to pharmacological stimulation and the plasma IGF I concentration in young adults with GHD of childhood onset and PSIS depend on the presence of additional pituitary deficiencies, reflecting a more severe defect of the hypothalamic-pituitary axis. This severity can also be assessed by the height of the anterior pituitary gland on magnetic resonance imaging and by the GH response to a GHRH test.

Thus diagnosis of GHD in the transition period must take into account the presence or absence of other pituitary deficiencies. The GH peak may be greater than 3 ng/ml and the plasma IGF I greater than 84 ng/ml if the GH deficit is isolated.

## Abbreviations

BMI: body mass index; GH: growth hormone; GHD: growth hormone deficiency; GHRH: growth hormone releasing hormone; IGF: insulin-like growth factor; PSIS: pituitary stalk interruption syndrome.

## Competing interests

The authors declare that they have no competing interests.

## Authors' contributions

MM participated in the conception and design, the acquisition of data and analysis. CT and J–CS carried out the immunoassays and the statistical analyses. RB directed the work and prepared the manuscript. All the authors have given final approval of the version to be published.

## Pre-publication history

The pre-publication history for this paper can be accessed here:



## Supplementary Material

Additional file 1 Table. Comparison of the 2 groups. Click here for file
