# Supplementary material for: Factors influencing the growth hormone peak and plasma insulin-like growth factor I in young adults with pituitary stalk interruption syndrome
Source: BMC Endocr Disord. 2008 Jul 11;8:7. doi: 10.1186/1472-6823-8-7 (PMC2474832; doi:10.1186/1472-6823-8-7)
Supplement: Supplementary file 1 [file 1472-6823-8-7-S1.doc]

Table 1. Comparison of the 2 groups

			At diagnosis							At last evaluation				
Patients	Sex	Associated deficiencies	Age years	Pituitary height mm	Height SDS	BMI SDS	GH peak ngm/l	GH after GHRH ng/ml	IGF I ng/ml (SDS)	Age years	GH peak ng/ml	IGF I ng/ml (SDS)	Testosterone ng/ml	Estradiol pg/ml
**GROUP 1 isolated GH deficiency**														
1, Y.P.	M	0	1.8	2.7	-2.1	-0.8	2.9	35.0	18 (- 3.8)	15.3	2.5	130 (-4.3)	5.8	
2, V.G.	M	0	3.3	2.0	-2.1	-2.0	4.8		18 (- 2.9)	15.3	2.7	291 (-2.1)	4.8	
3, C.B.	M	0	3.9	4.0	-1.7	-0.5	6.1		34 (- 2.5)	14.9	3.0	296 (-2.1)	1.7	
4, E.L.	F	0	5.0	4.0	-4.2	0.8	3.3		20 (- 4.9)	14.8	7.4	306 (-1.9)		30
5, B.L.	M	0	5.5	1.0	-2.9	-0.9	6.5		12 (- 5.2)	15.4	2.6	218 (-3.1)	6.7	
6, A.L.	M	0	8.3	4.0	-2.8	-0.4	4.4	26.6	49 (- 4.3)	18.3	6.4	155 (-3.9)	6.5	
7, G.F.	M	0	8.5	2.5	-3.7	-0.6	2.4	4.9	44 (- 4.4)	17.8	4.0	296 (-2.1)	7	
8, S.K.	F	0	9.2	4.0	-2.5	2.3	0.6		26 (- 4.9)	14.1	1.9	106 (-4.6)		37
9, D.C.	M	0	17.0	1.5	-4.7	-0.6	2.8		48 (- 3.8)	20.2	4.4	104 (-4.6)	4.5	
Mean ± SD			6.9±4.5	2.8±1.2	- 3.0±1.0	- 0.3±1.2	3.8±1.9	22.2±15	(- 4.1±0.9)	16.2±2.0	3.9±1.9	211±88 (-3.2±1.2)		
**GROUP 2 Multiple pituitary deficiencies**														
10, A.A.	M	TSH/ACTH/Gn	1.4	0.5	-2.2	0.0	5.3	13.1	27 (- 3.3)	15.8	0.0	59 (-5.2)	1.5	
11, S.F.	F	TSH/ACTH/Gn	2	2	-2.8	2.6	2.6		NA	15.9	0.0	87 (-4.8)		10
12, G.M.	M	TSH	2.7	2.0	-3.7	0.2	3	16.6	14 (- 3.1)	15.8	0.0	53 (-5.3)	5.6	
13, C.T.	M	TSH/ACTH/Gn	2.2	2.0	-2.5	-1.4	0.4	3.2	NA	14.7	0.0	44 (-5.4)	0.63	
14, T.L.	M	TSH/ACTH/Gn	3.3	0.5	-3.2	-1.4	1.5	11.6	4 (-3.3)	16.3	0.0	56 (-5.3)	0.74	
15, M.A.	M	TSH/ACTH/Gn	4.0	4.0	-0.8	0.2	1		24 (- 4.8)	15.0	0.0	78 (-5.0)	1.4	
16, H.G.	F	TSH/ACTH/Gn	4.2	1.0	-0.9	6.8	0	0.3	28 (- 4.6)	12.4	0.0	69 (-5.1)		7
17, C.M.	M	TSH/Gn	4.3	3.0	-6.2	-1.6	0	6.0	3 (- 5.5)	16.7	0.0	20 (-5.7)	0.06	
18, S.D.	F	TSH	4.4	1.0	-2.6	0.8	2.5		14 (- 5.1)	15.8	0.9	275 (-2.3)		13
19, E.D.	F	TSH	4.4	2.0	-2.1	-1.3	1.4	10.9	11 (- 5.2)	15.7	0.9	133 (-4.2)		12
20, R.D.	F	TSH/ACTH/Gn	9.4	0.5	-5.2	1.4	0.7	10.4	NA	17.6	0.0	55 (-5.3)		<10
21, F.D.	F	TSH/ACTH/Gn	14.0	1.0	-4.5	-2.0	0.7	1.8	5 (- 4.6)	18.9	0.7	17 (-5.8)		29
Mean ± SD			4.7±3.6	1.6±1.1	- 3.1±1.6	0.4±2.4	1.6±1.5	8.2±5.6	(- 4.4±0.9)	15.9±2.0	0.2±0.4	78±69 (-5.0±0.9)		
Group 1 compared to 2			NS	P=0.03	NS	NS	P<0.02	NS	NS	NS	P=0.0001	P<0.002(ng/ml) P<0.002(SDS)		

TSH: thyroid stimulating hormone
ACTH: adrenocorticotropin hormone
Gn: gonadotropin hormone
